# Solvent and HEMA Increase Adhesive Toxicity and Cytokine Release from Dental Pulp Cells

**DOI:** 10.3390/ma12172750

**Published:** 2019-08-27

**Authors:** Helder Massaro, Lígia F. A. Zambelli, Auriléia A. de Britto, Rodolfo P. Vieira, Ana P. Ligeiro-de-Oliveira, Denise C. Andia, Marcelo T. Oliveira, Adriano F. Lima

**Affiliations:** 1 Department of Endodontics, Paulista University, Rua Doutor Bacelar, 1212, Sao Paulo 04026-002, Brazil; 2 Department of Restorative Dentistry, University Nove de Julho (UNINOVE), Sao Paulo 01504-001, Brazil; 3 Post Graduate Program in Biophotonics Applied to Health Sciences, University Nove de Julho (UNINOVE), Sao Paulo 01504-001, Brazil; 4 Post-graduation Program in Bioengineering and in Biomedical Engineering, Universidade Brasil, Rua Carolina Fonseca 235, São Paulo 08230-030, Brazil; 5 Department of Sciences of Human Movement and Rehabilitation, Federal University of São Paulo (UNIFESP), Avenida Ana Costa 95, Santos 11060-001, Brazil; 6 Brazilian Institute of Teaching and Research in Pulmonary and Exercise Immunology (IBEPIPE), Rua Pedro Ernesto 240, São José dos Campos 12245-520, Brazil; 7 School of Medicine, Anhembi Morumbi University, Avenida Deputado Benedito Matarazzo 4050, São José dos Campos 12230-002, Brazil; 8 Dental Research Division, Paulista University, Rua Doutor Bacelar, 1212, Sao Paulo 04026-002, Brazil; 9 Department of Restorative Dentistry, Ibirapuera University, Av. Interlagos, 1329-Chácara Flora, São Paulo SP 04661-100, Brazil

**Keywords:** polymers, cytotoxicity, inflammation, pulp biology, biocompatibility, adhesives

## Abstract

The aim of the present study was to evaluate the effect of the hydroxyethyl-methacrylate (HEMA) concentration and solvent content of dental adhesives on cell viability and cytokine (IL-1b, IL-6, IL-10, TNF-α) release by human dental pulp cells (HDPCs). HDPCs were obtained from fresh extracted human third molars. Experimental adhesives were prepared containing different concentrations of HEMA (0%, 10%, and 20%) with and without solvent (ethanol 10%). Cylindrical specimens were immersed on culture medium during 24 h to obtain the extracts. The cells were incubated with extracts (culture medium + components leached from the adhesives) of different adhesives, and cell viability and cytokine release were evaluated after 6 and 24 h of exposure. Adhesives containing HEMA promoted high cell viability reduction after 6 h of exposure; but after 24 h, the results were similar to the ones found among control group cells. These effects on cell viability were prominently increased with the addition of solvent. Although IL-1b release was not affected by exposure to eluates, other cytokines (IL-10, IL-6, TNF-α) were modulated by the different experiment conditions, directly influenced by the HEMA concentration and presence of solvent. Higher HEMA concentrations, combined with the presence of solvent, can promote significant reduction on HDPC viability, increasing the release of anti- and pro-inflammatory mediators.

## 1. Introduction

Resin materials are applied in several dental restorative procedures such as surface sealers [1], cementation of intraradicular posts [2], repair of restorations [3,4] as well as bonding direct and indirect restorations [5,6,7]. The dental adhesives are agents used to promote the bonding between the dental substrate and the resin materials. Due to the direct application on the dental structure, the dental material’s should be evaluated regarding its biocompatibility, including cytotoxic effects [8,9,10,11] or in vivo evaluations [12,13], to avoid toxic materials being used which can put patients at risk of health problems. In situations like bonding to deep cavities, the resin monomers present in adhesive agents can diffuse through the dentinal tubules of the thin remaining dentin and reach the dental pulp, triggering cell and tissue responses such as inflammation processes [14,15]. This may induce processes such as a transitory hypersensitivity after restorative procedures, and/or could require endodontic treatment in some cases with non-remission of symptoms. 

The inflammation is initiated by intracellular signalling cascades, resulting in the release of anti- and pro-inflammatory mediators, coordinating the immune response [16], and may be triggered by the damage caused by the monomers to the dental pulp cells (fibroblasts, odontoblasts, and macrophages). Cytokines such as the IL-1, the IL-6 and the TNF-α can kick start the inflammatory process by inducing alterations on the tissue, such as vasodilatation and defence cell recruitment [17]. In addition, anti-inflammatory cytokines as the IL-10 can regulate this process, promoting a balance between tissue damage and response [17]. 

As described, the monomers are toxic agents that can promote cell damage and consequently, undesirable side effects on restorative procedures. Notwithstanding the hydroxyethyl methacrylate (HEMA) not being the most toxic monomer used [18], the concern about this agent is based on its low molecular weight, which allows such compound diffusion through the dentinal tubules, reaching the pulp tissue [19,20]. HEMA influences cell function and cytokine release [21], which can modulate the tissue response facing an aggression promoted by exposure to this agent. 

Another aspect that should be considered in dental adhesives related to the toxic implication to the dental pulp cells is the presence of solvent. Considering its inexorableness in view of the wet nature of dentin, the solvent can increase the solubility of dental adhesives [22,23], increasing the release of unreacted monomers to the aqueous environment. Consequently, this may enhance the toxic effects of resin adhesive agents.

Despite not being fully elucidated, several studies report the toxic effects of monomers to different cell types, demonstrating early injuries at membrane level and DNA-deep viability reduction [21,24,25]. These studies are important, as they provide the mechanisms regarding toxicity and influence on cell functions [21,24,25]. However, most of the studies evaluate the monomers isolated, which does not correspond to a clinical environment. This may lead to equivocated conclusions, considering that the toxicity of a specific monomer could not be directly implicated in co-polymer toxicity increase. When mixed with other monomers and co-polymerized, the effect of a specific monomer on cells may be different due to the curing reaction and interaction among the agents, and/or entrapment of unreacted monomers on the polymer chain, preventing their toxic effect. Therefore, the aim of this study was to evaluate the influence of experimental solvated and non-solvated adhesives containing different concentrations of HEMA and their viability and cytokine release (IL-1, IL-6, TNF-α, and IL 10) from human dental pulp cells (HDPCs).

## 2. Materials and Methods

### 2.1. Cell Culture

Human dental pulp cells (HDPCs) were obtained from fresh extracted human third molars (Ethics Committee Approval from Paulista University #2.961.606, São Paulo, Brazil). The teeth were extracted, and the coronal part was sectioned on the cement-enamel junction using a sterilized diamond disc under water-cooling. The pulp tissue was removed using a dentin excavator and immersed in culture medium. After this, the tissue was immersed in collagenase type 1 (3 mg/mL; Sigma-Aldrich, St. Louis, MO, USA). After 2 h, the resulting cells were cultured in Dulbecco’s Modified Eagles’ Medium (DMEM, SIGMA Chemical Co., St. Louis, MO, USA) complemented with 10% foetal bovine serum (FBS, Cultilab, Campinas, São Paulo, Brazil), supplemented with 100 IU/mL of penicillin, 100 mg/mL streptomycin and 2 mmol/L glutamine (GIBCO, Grand Island, NY, USA) until obtaining the cell number needed to perform the experiments. 

### 2.2. Experimental Groups

The experimental adhesives were prepared, and the groups distributed as presented in Table 1, according to the HEMA (Esstech Inc., Essington, PA, USA) concentration and presence of solvent (ethanol, Merck KGaA, Darmstadt, Germany). In addition to these components, the adhesives included bisphenol glycidyl methacrylate (Bis-GMA, Esstech Inc., Essington, PA, USA) and triethylene glycol dimethacrylate (TEGDMA, Esstech Inc., Essington, PA, USA). For all formulations, the used initiator system was the camphorquinone (CQ, Esstech Inc., Essington, PA, USA) 0.4% wt., and co-initiator: dimethylaminoethyl amine benzoate—0.8% wt. (EDAB, Sigma-Aldrich Inc., St. Louis, MO, USA). All components were blended and homogenized for 1 h at room temperature (25 °C) with a magnetic stirrer (2000 rpm). The composition of experimental adhesives within the respective group is listed in Table 1. 

### 2.3. Preparation of Eluates

To obtain adhesive eluates, samples (5 mm diameter, 1 mm thick) were prepared using a silicon mould. After insertion of adhesive in the mould, the resin was covered by a glass slide (0.1 mm thick) to avoid the formation of the oxygen-inhibited layer. Then, the samples were light-cured for 20 s using a light-emitting diode unit (LED, Demi; 1200 mW/cm^2^, Kerr Company, Orange, CA, USA), with the LED tip in contact with the glass-slide. Then, each specimen was individually placed in wells of a 24-well sterile plate containing 1 mL of DMEM with no foetal bovine serum (FBS) and maintained in an incubator at 37 °C for 24 h. Finally, the eluates were collected and applied on the HDPCs and kept in contact for 6 h. For the control group (G0), the cells were cultured on fresh culture medium (no FBS) at the same time it occurred with the eluates, and kept for the same period, to simulate the experimental condition. 

### 2.4. Cell Viability (MTT Assay)

For the experimental and control groups (n = 6), the cell viability was evaluated using MTT assay by measuring the succinic dehydrogenase (SDH) activity, evaluating the mitochondrial activity of cells by the methyl tetrazolium reaction. The assays were made in triplicate at three independent experiments. As previously described, the cell metabolism was evaluated 6 and 24 h after exposure to the eluates [26]. The cell viability was obtained in absorbance of SDH activity and transformed in percentages considering the control group (DMEM) as 100%.

### 2.5. Quantification of Cytokines Released from Dental Pulp Cells in Culture

Cytokines levels were determined in culture medium in contact with the HDPCs after eluates exposure (n = 6; 6 and 24 h). The timepoints used were chosen based on previous study [27]. The analyses were performed according to the manufacturer’s instructions using ELISA kits from Biolegend (San Diego, CA, USA). The plates were read with a spectrophotometer (SpectraMax i3; Molecular Devices, CA, USA) at a wavelength of 450 nm. The concentrations of the cytokines in the samples were calculated from the standard curves obtained from the recombinant cytokines [28]. Results were expressed as pg/mL of cytokine. The assays were made in triplicate at three independent experiments for every sample with standard curves for IL-1b, IL-6, IL-10, TNF α. The detection limits of each cytokine were 1–125 pg/mL for IL-1b; 4–500 pg/mL for TNF- α and IL-6, and 2–250 pg/mL for IL-10.

### 2.6. Statistical Analysis

Prior to statistical analysis, the data was analysed for normality and homogeneity of variance. Cell viability and cytokine release were analysed using one-way ANOVA. The Tukey’s test was applied when needed. Statistical analysis was carried out using the SAS 9.1 statistical software (SAS Institute, Cary, NC, USA) with a confidence interval of 95%. 

## 3. Results

### 3.1. Cell Viability and Cytokine Release (6 h)

**Figure 1:**
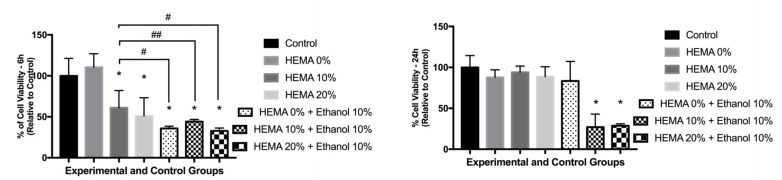
Cell viability of dental pulp cells after exposure to extracts (6 h) of different adhesive models. The percentages were obtained considering the control group (DMEM) as 100%. * indicate statistical difference to the control group (*p* < 0.001). # indicate difference of HEMA10% to other experimental groups—# *p* < 0.001; ## *p* < 0.05. ANOVA one-way and Tukey test (α = 0.05).

After exposure, the toxic effects were more evident, with lower cell viability observed for the groups exposed to eluates of solvated adhesives (Figure 1). The non-solvated adhesives containing HEMA (10% and 20%) had intermediary cell viability, whilst the non-solvated adhesive without HEMA showed higher cell viability, similar to the control group (*p* > 0.999). 

**Figure 2:**
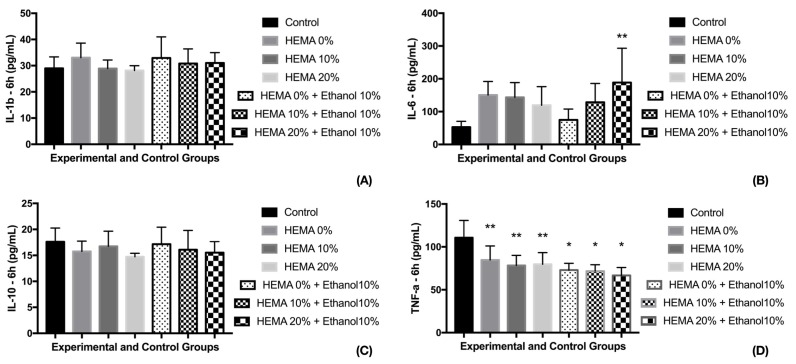
Cytokine release 6 h after exposure to different adhesive models. * indicate statistical significant difference to control group—* *p* < 0.001; ** *p* < 0.05. (**A**) For IL-1b there was not observed statistical differences among control and experimental groups (**B**) Only Hema 20% + Ethanol presented statistical difference to control. The other experimental groups had intermediary results, similar to control and HEMA 20% + ethanol. ANOVA one-way and Tukey test (α = 0.05). (**C**) The experimental and control groups were statistically similar (**D**) Control group had the highest TNF-a release compared to the other groups. The experimental groups presented similar TNF-a release.

For the cytokine release, this relation was not perfectly elicited (Figure 2). After 6 h, no changes were observed in IL-1b, IL-10 (*p* = 0.4922 and 0.5330). However, for IL-6, the solvated adhesive containing 20% HEMA (G6) presented the higher cytokine release, with control group presenting the lowest values. The other experimental groups had intermediary release, similar to control and G6. For TNF-α, the control group presented the highest means after 6 h, with all experimental groups with similar results.

### 3.2. Cell Viability and Cytokine Release (24 h)

The most prominent reduction on cell viability was observed for the solvated adhesive containing HEMA 10 and 20%, statistically different of the other groups (*p* < 0.0001) (Figure 1). For this period (24 h), the other tested adhesive models did not influence cell viability, which was similar to the control groups. 

**Figure 3:**
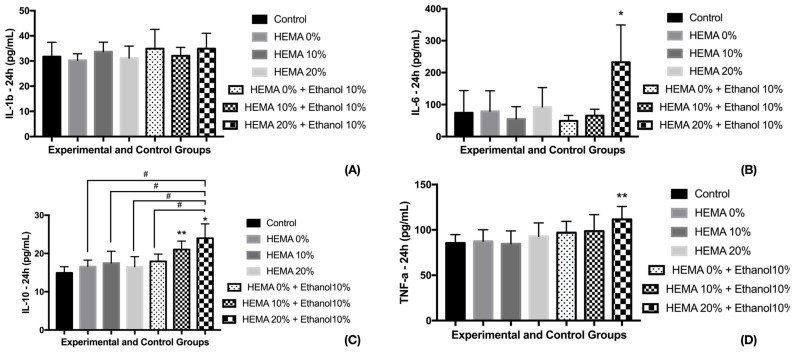
Cytokine release 24 h after exposure to different adhesive models. * indicate statistical significant difference to control group—* *p* < 0.001; ** *p* < 0.05. (**A**) For IL-1b, none differences were observed after 24h of exposure. (**B**) The group containing HEMA 20% with ethanol promoted the higher release of IL-6, statistically superior than the other experimental and control groups. (**C**) # indicate statistical difference of experimental groups and HEMA 20% + ethanol—# *p* < 0.001. (**D**) Groups with 0% and 10% HEMA containing ethanol were similar to control group and HEMA 20% + ethanol. ANOVA one-way and Tukey test (α = 0.05).

For IL-1b, the experimental groups had similar release to the control group (no exposure to eluate) (Figure 3; *p* = 0.6595). For IL-6, G6 (HEMA 20% + solvent) presented the highest release, whereas the other experimental groups and control presented similar mean values. G6 had the highest means of IL-10 as well, decreasing the amount released according to HEMA reduction and the absence of solvent, with the control group presenting the lowest values. 

For TNF-α, the control group and groups G1 (no HEMA) and G2 (HEMA 10%) presented the lowest releases. G6 had the highest means whilst the other groups (G3, G4, and G5) presented intermediate release, being similar to control group and to G6.

## 4. Discussion

HEMA is a monomer that is widely used on dental adhesives due to its ability to reduce monomer mixture viscosity, improving the adhesive system wettability [29,30]. However, due to its low molecular weight, HEMA can diffuse through the thin dentin barrier of deep cavities [19,20], reaching the dental pulp cells, promoting deleterious effects to the pulp tissue due to its toxicity [31]. Another important fact regarding the adhesive agents that should be considered is the presence of solvent. This modulates the properties of such materials, influencing the polymer formation, and consequently, the water solubility [22,23,32], which may increase the toxicity of the adhesive agent.

Adhesives with no solvents are commercially presented as the third step on the etch-and-rinse or second step of self-etch adhesives processes, following primer application. The use of solvated adhesives without the use of a hydrophobic adhesive is observed when using simplified systems like the one-bottle etch-and-rinse, or the self-etching and multi-mode adhesives [33]. Agents such as acetone, ethanol, and water are the most used solvents on dental adhesives, with concentration ranging from 20% to 30%. Despite the required step of solvent evaporation, the complete removal of this agent is not feasible [32], which justifies the evaluation of solvated adhesives using 10% ethanol in the present study, representing the remaining solvent on the adhesive after the solvent evaporation step. 

The obtained cell viability results demonstrate that the higher the amount of HEMA, the higher the toxic effects to the cells. Nevertheless, this toxicity is temporary, with increase of cell viability 24 h after exposure to the non-solvated adhesives. In addition, HEMA toxicity is prominently increased when solvent was added to the experimental adhesives, demonstrating that the modulation of adhesive properties by the solvent, such as water sorption and solubility, can directly influence the toxicity to the HDPCs, as previously shown by some studies [23,32,34,35].

Ethanol can increase the water solubility of dental adhesives [36], and the increasing of this property may allow the leaching of higher amounts of toxic components. A previous study has demonstrated the high cytotoxicity of universal adhesives [8]. Despite different methodology and cell lines used, which can explain some differences in the results of the MTT assay, universal adhesives are single-bottle agents, presenting high content of solvent in its composition. As demonstrated in this study, solvent is a component that can increase remarkably the toxicity of the adhesives. This can be one of the most important reasons for the obtained results on the study cited [8].

Bis-GMA, TEGDMA and HEMA, components present on the tested adhesive models, were probably released in higher concentration than the non-solvated adhesives, which may have promoted more expressive toxic effects. Bis-GMA, one component of the model adhesives tested, was shown to induce COX-2 expression and have PGE2 production via reactive oxygen species (ROS) production, leading to dental pulp cells damage [37]. In addition, Bis-GMA is the most toxic monomer used in bonding agents [38], and even in reduced concentrations, it may cause damage to pulp cells, such as DNA lesions, and also oxidative stress due to the generation of ROS and GSH depletion [24,39]. The damage caused by ROS and the epoxy-compound metabolic intermediary of the Bis-GMA are possibly involved in cell apoptosis after exposure to this resin monomer, as a result of the down regulation of cdc2, cdc25C, and cyclinB1 [24], important genes that regulate cell cycle progression and apoptosis [40].

TEGDMA and HEMA promote oxidative DNA damage caused by reactive oxygen species (ROS) [21]. After cell exposure to these monomers, high levels of 8-oxoguanine (8 oxoG), a promoter of oxidative DNA lesion, were noticed [25]. Although the effects observed in the present study may be related to the different compounds present on the model adhesives, the direct influence of an increased amount of HEMA promoting higher cytotoxicity and, more relevantly, the presence of solvent, were clearly observed, considerably increasing the toxic effects. 

The aggression to the cells cannot only cause cell viability reduction, but can also promote a release of inflammatory mediators. IL-1 can start the inflammatory reaction, stimulate T-cell proliferation, and stimulate the release of histamine from mast cells [17,41]. The major cellular sources of IL-1 are fibroblasts, mononuclear phagocytes, and T and B lymphocytes [17]. Despite its importance in the inflammatory process, IL-1 release was not influenced by the experimental conditions of this study, in neither of the evaluated periods. 

TNF-α, an important cytokine that can stimulate collagenase production and IL-6 synthesis and, like the IL-1, initiate the inflammatory process [17,41], was influenced by the adhesives tested in the present study. After 6 h, the experimental groups showed reduced release TNF-α compared to control. However, after 24 h, the solvated adhesives containing HEMA 20% presented the highest release, with groups 3, 4, and 5 presenting an intermediary cytokine release level. 

The IL-6 higher release promoted by solvated resin containing HEMA 20% was observed after 6 and 24 h of exposure, with the other experimental groups presenting intermediate (6 h) or similar release to the one noticed in control (24 h). IL 6 is produced by some cells including T-cells and fibroblasts. IL-6 acts as a growth factor for mature B-cells and it is involved in T-cell activation and differentiation [17,41]. The release of IL-6 in few hours (6 h) after exposure can indicate that an inflammatory process can be initiated just after the bonding procedure to deep cavities and diffusing of unreacted/leached monomers to the dental pulp. Based on these results, this inflammatory process seems to be more prominent when solvated adhesives with a high amount of HEMA are used. 

After aggression, not only pro-inflammatory but also immuno-modulatory mediators, such as IL-10, may be released [42]. As observed, after 6 h of exposure, the dental pulp cells presented a similar release of the cited cytokine. However, after 24 h, a significantly high amount of IL-10 was observed on groups of solvated adhesives containing 10% or 20% HEMA. The release after 24h exposure could be credited to the IL-6 release, which stimulates the release of potent anti-inflammatory mediators such as IL-10 [43]. IL-10 inhibits the release of inflammatory cytokines, and inhibits the formation of the inducible NO-syntheses (iNOS) and consequently the formation of NO [44], which can induce cell apoptosis. This result indicates that, despite the aggression and release of pro-inflammatory cytokine at early stages (6 h), the cells are able to release immuno-modulatory mediators aiming to control the inflammatory process promoted by the leached monomers. 

One limitation of the present study is the non-quantification of the released components after immersion. However, this release was well demonstrated in several studies [45,46], and based on previous results, the time of immersion was stipulated as well as the discussion was supported. Despite the toxicity of other tested experimental adhesive components (Bis-GMA and TEGDMA), the main focus of this study was to evaluate the influence of HEMA and solvent on adhesives and these effects on HDPCs, as these compounds can diffuse through the dentinal tubules, reaching the dental pulp [47]. As demonstrated, the toxicity of adhesives can be modulated by the presence of HEMA. However, the effects are relevantly increased by the presence of solvents, probably due to the adhesive solubility increase, consequently leaching unreacted monomers. The bonding procedures to address deep cavities are critical and should be performed carefully due to the possibility of unreacted monomers diffusion to the pulp tissue, which makes the study of variables and protocols necessary for better understanding the consequences to dental pulp cells.

The development of new materials on dentistry is fast, focusing on the improvement of material properties [48,49,50,51] and patient wellbeing. With this, studies evaluating the toxic effects of new and existing materials are important to provide a safe and predictable treatment on the most diverse dental specialties. Based on this, future studies focusing on biomaterial evaluation are important to maintain the safe application and development of the dental products. 

## 5. Conclusions

HEMA presents a dose-dependent toxic effect on pulp cells, which can be increased by the addition of solvent. These results highlight the previous statement that not only the presence of HEMA or other toxic monomers can indicate the toxicity of a resin material. The evaluation of the interaction between the components in a co-polymerization, in different simulated clinical conditions is important to understand the materials and its indications. The presence of solvent not only significantly reduced cell viability, but also increases cytokine release, indicating that solvated adhesives can be more harmful than non-solvated ones when used in deep cavities with thinner dentin barrier separating the restorative procedure from the pulp tissue. 

## Figures and Tables

**Table 1:** Experimental groups and their respective monomer composition.

Group	Composition (%)			
	Bis-GMA	TEGDMA	Ethanol	HEMA
G1	50	50	−	−
G2	45	45	−	10
G3	40	40	−	20
G4	45	45	10	−
G5	40	40	10	10
G6	35	35	10	20
G0—Control (without treatment)				
